# Supplementary material for: Molecular Response of Meyerozyma guilliermondii to Patulin: Transcriptomic-Based Analysis
Source: J Fungi (Basel). 2023 Apr 30;9(5):538. doi: 10.3390/jof9050538 (PMC10219284; doi:10.3390/jof9050538)
Supplement: Supplementary file 1 [file jof-09-00538-s001.zip › jof-2351183-supplementary.pdf]

Table captions

Table. S1：Primers used in RT-qPCR (*M. guilliermondii*).

Gene ID	Gene Name	Forward primer (5' to 3')<br>Reverse primer (5' to 3')
PGUG_00994	*NOP53*	TCACAGTCACACTCGTGCCAAAG<br>TGCCACCCACCACCTCATCATC
PGUG_01741	*MRR1*	AACCCATACCGCAACCACAACG<br>ACAAGCCCAAATCTCGCCTCATG
PGUG_01881	*SPCC24B10.20*	TGGTCAATCGAAGGCTGCTCT<br>TCGTGGCCCATATCGGTTCC
PGUG_05287	*YML131W*	TCTGCTGCTTCTGGGGCTACC<br>GACGCAAACATCGGCACCAATAC
PGUG_01077	*SPCC663.08c*	CACCGCAGCACACACCATCC<br>GACCAATTCCACGAGAAGCACCAG
PGUG_04552	*GRP2*	AAGGCAGCGTGGGAGTTTGTTG<br>TCAGCGGTCATGTTGAGCGTTC
PGUG_05192	*YPR022C*	GGTGTTCGTGGCCTGATTGTGG<br>TGGTTCTGTCTTGTTCTCGTGCTG
PGUG_04009	*YJR096W*	GATCCACTCGCCTCAATCGAACAG<br>TCCAATCCGTCCCATTCCAACAAC
PGUG_05193	*SPCC663.09c*	CTGATTGCGGCTCGTTAAACTTGG<br>GCTCCAACTGTCTCGCCAACTC
PGUG_01005	*GSH2*	TTCGGAACTTGCTCGTGGTCTTG<br>CGCTCGCCAGGTTGAACAATTAC
PGUG_03271	*MDR1*	GGTCAATGGCAGCAAAACTCTTCG<br>CTTCGGCACCAGGAGTGTAGATTG
PGUG_00888	*YHK8*	GACGACGGCAACGACAAGGAG<br>AAGCTAGGGACCATGCTGAGGAG
PGUG-05383	β-tublin	CCAATTCCTGGCTCCGCTTACG<br>CTGTCCTACTTCCGCACCCTTTTC

Table. S2：Comparison of clean data and reference genome.

Treatment	samlpes	reads aligned (%)	exonic (%)	intronic (%)	intergenic (%)
CK	CK-1	96.69	85.09	0.02	14.89
	CK-2	96.58	84.87	0.02	15.11
	CK-3	96.29	84.37	0.02	15.62
Pat	Pat-1	96.46	85.70	0.02	14.28
	Pat-2	96.44	85.09	0.02	14.89
	Pat-3	96.78	84.90	0.02	15.09

Table. S3：Number of annotated DEGs.

DEG Set	DEGs Number	Swiss-Prot	GO	KEGG	COG	KOG	Pfam	NR
CK_vs_Pat	348	281	275	160	105	194	279	323
